# Inulin does not affect trimethylamine N‐oxide formation in mice with a high‐fat diet combined with choline and L‐carnitine

**DOI:** 10.1002/fsn3.4420

**Published:** 2024-09-15

**Authors:** Xin Wang, Xiaoyi Hu, Weiwei He, Jun‐Yi Yin

**Affiliations:** ^1^ State Key Laboratory of Food Science and Resources, China‐Canada Joint Laboratory of Food Science and Technology (Nanchang) Nanchang University Nanchang China

**Keywords:** choline, functional genes, gut bacteria, L‐carnitine, metagenomics

## Abstract

Emerging evidence suggests that gut bacteria‐derived trimethylamine N‐oxide (TMAO) elevates the risk of cardiovascular disease, and dietary fiber holds the potential to attenuate TMAO formation. However, the effectiveness of dietary fiber, such as inulin, in inhibiting TMAO formation remains controversial. Therefore, this study investigated the effect of inulin supplementation on TMAO formation in mice with high TMAO levels induced by a high‐fat diet (HFD) combined with choline and L‐carnitine. Results showed that HFD treatment significantly elevated blood TMAO concentrations and increased the abundances of TMAO formation‐associated gut bacteria, as well as the abundances of functional genes responsible for TMA formation. While the supplementation of choline and L‐carnitine greatly enhanced blood trimethylamine (TMA) and TMAO levels, inulin supplementation did not significantly affect TMAO levels and had limited impact on TMA‐associated gut bacteria, except for *Desulfitobacterium hafniense*.

## Graphical abstract

This study investigated the effects of inulin supplementation on trimethylamine N‐oxide (TMAO) levels in mice with elevated TMAO. The high‐fat diet (HFD) treatment resulted in increased blood TMAO concentrations and a higher abundance of gut bacteria associated with TMAO formation. However, inulin did not affect TMAO levels or the gut bacteria linked to TMAO formation in mice fed a high‐fat diet combined with choline and L‐carnitine.

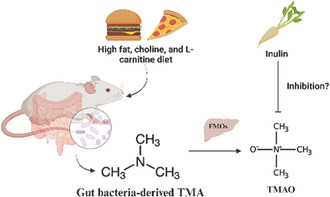

## 1 INTRODUCTION

Recent studies have suggested a negative effect of elevated plasma trimethylamine N‐oxide (TMAO) levels on human health, particularly cardiovascular disease (Jian et al., 2023; Zheng & He, 2022). The precursors of TMAO, such as choline and L‐carnitine, are widely found in food, particularly in red meats like pork and beef (Delgado et al., 2021). When choline and L‐carnitine escape absorption in the upper gastrointestinal tract, they reach the distal colon where they are metabolized by intestinal bacteria into trimethylamine (TMA). Subsequently, TMA is absorbed and converted into TMAO by the hepatic enzyme flavin‐containing monooxygenase 3 (FMO3) in the liver. Therefore, excessive intake of L‐carnitine and choline is considered to be a crucial cause of elevated TMAO levels.

Since meat products are rich in choline and L‐carnitine, excessive meat product intake is closely related to the increased risk of cardiovascular disease. Koeth et al. (2013) conducted a study comparing changes in gut microbiota and TMA levels in omnivores and vegetarians after ingestion of isotope‐labeled L‐carnitine (Koeth et al., 2013). The results showed that initially, there was no significant difference in fecal TMA levels between the two groups. However, after consuming the same amount of L‐carnitine, the fecal TMA levels in omnivores were significantly higher than in vegetarians. Subsequently, the researchers isolated several bacterial strains from the feces of omnivores that can convert L‐carnitine into TMA (Koeth et al., 2013).

In addition to being rich in choline and L‐carnitine, intake of high‐fat content in animal products may also contribute to increased TMAO levels. A recent study conducted by Yoo et al. (2021) unveiled that a high‐fat diet (HFD) was correlated with elevated TMAO levels. This elevation is attributed to mitochondrial damage in the colonic epithelium, impairing the conversion of choline to TMAO (Yoo et al., 2021). However, there is currently a lack of systematic analysis on how HFD treatment affects the abundance of functional microbial communities related to TMA formation.

Dietary fiber is a polysaccharide that cannot be digested and absorbed in the proximal gastrointestinal tract. Instead, it passes into the distal colon, where it is then broken down into short‐chain fatty acids (SCFAs), such as acetate, propionate, and butyrate, through the interaction and feeding by intestinal microorganisms, thereby altering gut microbiota composition (Vinelli et al., 2022; Zhang, Xie, et al., 2021). In recent years, some studies have extensively studied the inhibitory effects of different dietary fibers on TMA/TMAO formation (Lamichhane et al., 2016; Li et al., 2017). However, the efficacy of dietary fiber in inhibiting TMAO remains controversial. For instance, several studies have shown that dietary fibers such as inulin do not significantly affect TMAO levels (Baugh et al., 2018; Xiong et al., 2023). However, whether inulin supplementation has an inhibitory effect on diet‐induced high blood TMAO levels has not yet been studied.

Therefore, this study examined the impact of inulin supplementation on blood TMAO levels under conditions of high‐fat, high‐choline, and high carnitine intake, simulating a Western diet rich in animal products. Furthermore, metagenomics was employed to explore the biological mechanisms by which diet intervention affects TMAO levels through the analysis of functional gene levels. These findings may provide a theoretical reference for reducing TMAO levels elevated by the high consumption of animal product‐based foods.

## 2 METHODS AND MATERIALS

### 2.1 Materials

A highly soluble inulin (>86%, chain length ranges between 2 and 70) was purchased from BENEO (Orafti® HSI, BENEO Asia Pacific Pte. Ltd., Singapore). L‐Carnitine (98%; CAS # 541‐15‐1) and choline chloride (AR, 98%; CAS # 67‐48‐1) were purchased from Shanghai Aladdin Biochemical Technology Co. Ltd. (Shanghai, China). Trimethylamino‐d9 N‐oxide (98%; CAS # 1161070‐49‐0) and acetonitrile (chromatographic grade) were purchased from Merck‐Sigma (Shanghai, China). Trimethylamine‐d9 hydrochloride (98%; CAS # 18856‐86‐5) was purchased from Shanghai ZZBio Co., Ltd. (Shanghai, China).

### 2.2 Animal study

Twenty specific pathogen‐free (SPF) male C57BL/6J mice, aged 4 weeks, were obtained from GemPharmatech Co., Ltd. (Nanjing, China). The mice were acclimatized for 1 week in an SPF environment, receiving a standard diet (AIN‐76A) with ad libitum access to diet and tap water. Environmental conditions were maintained at a stable temperature of 25 ± 3°C, relative humidity of 50%–60%, and a 12‐h light/dark cycle.

**FIGURE 1:**
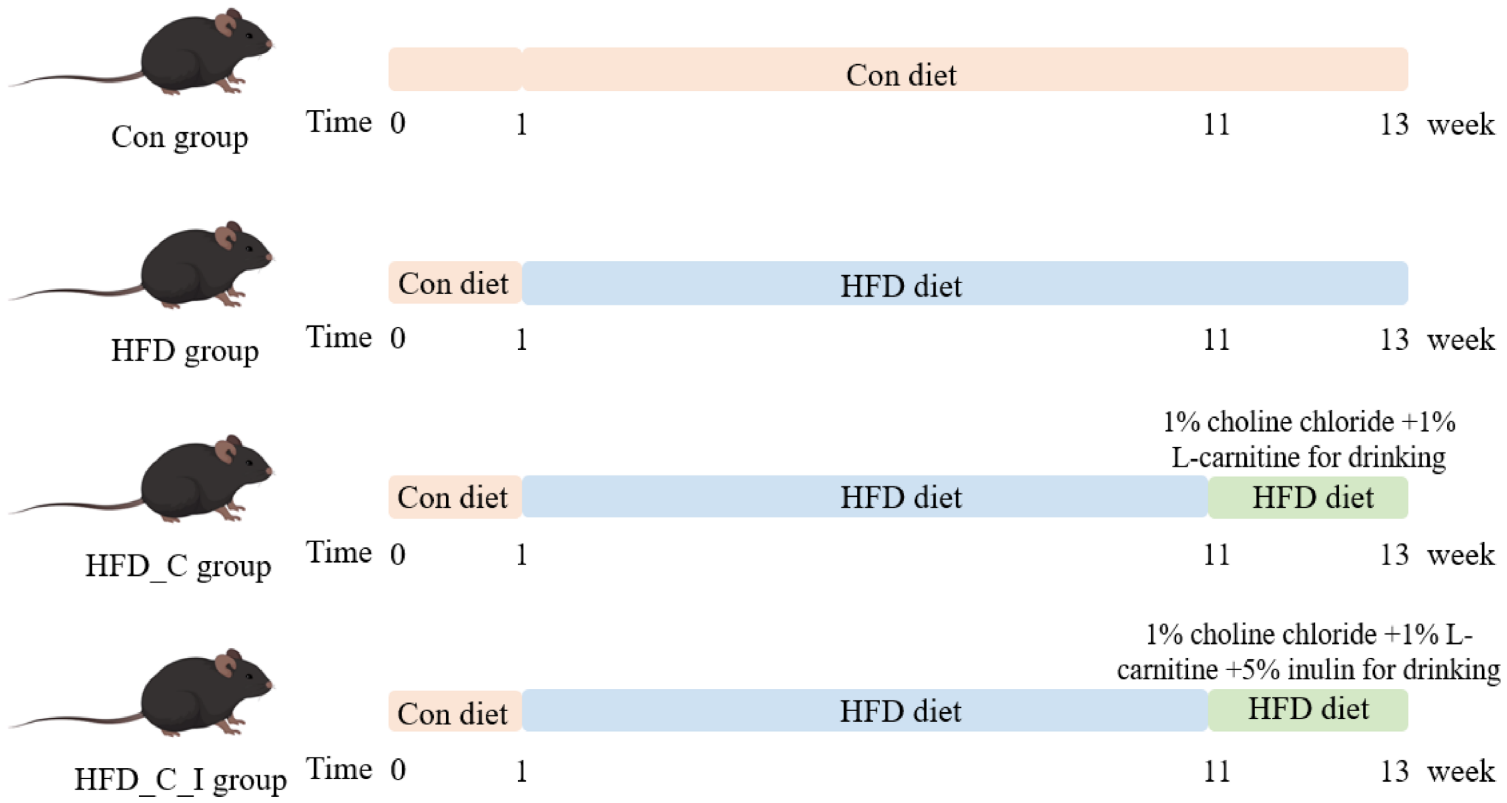
Experimental model scheme with four dietary groups.

After 1 week of adaptation, the experimental mice were randomly divided into four groups (*n* = 5). One group still received a standard control diet (Con), while the other three groups were provided with a high‐fat diet (HFD) fortified with 24.5% (w/w) lard oil. The nutritional compositions of the control diet and HFD are detailed in Table S1. As shown in Figure 1, after 11 weeks of HFD treatment, the drinking water for two of the HFD groups was replaced with solutions containing 1% (w/w) choline and 1% (w/w) L‐carnitine (HFD_C) or 1% (w/w) choline, 1% (w/w) L‐carnitine, and 5% (w/w) inulin (HFD_C_I). These treatments continued for an additional 2 weeks. Diet and water intake were recorded to calculate the consumption of choline and L‐carnitine. After dietary intervention, blood samples were collected from the mice via orbital bleeding, and fecal samples were collected from the rectum, then stored at −80°C. At the end of the experiment, the mice were euthanized by decapitation.

This animal study received authorization from the Science and Technology Department of JiangXi Province and the Institutional Animal Care and Use Committee of Nanchang University (Permission No.: SYXK [Gan] 2021‐0004). The study was conducted in strict adherence to China's Guidelines on Welfare and Ethical Review for Laboratory Animals.

### 2.3 Metabolite detection

Isotope labeling mass spectrometry quantitative method was employed to detect variations of TMA and TMAO concentrations in serum and feces.

A total of 20–40 mg fecal samples were weighed, and 200–400 μL acetonitrile and two small grinding steel balls were added for grinding extraction. The feces were centrifuged at 16,000× *g* for 15 min at 4°C, and the supernatant was absorbed to obtain the fecal extract. Standard substances, such as Trimethylamine‐d9 hydrochloride and Trimethylamino‐d9 N‐oxide, were dissolved into 1 mg/mL as reserve liquid using mass spectrum acetonitrile, and 10 μL of each was mixed and diluted to 10 mL as mixed internal standard (1 μg/mL). The concentrations of choline and L‐carnitine were determined using the standard curve method, while glycine betaine was presented as a relative concentration normalized to the choline signal.

A volume of 10 μL mixed internal standard solution (1 μg/mL), 10 μL serum or fecal extract, and 45 μL of 15 mg/mL ethyl bromoacetate (EBA) solution (acetonitrile solution with 1% ammonia) was mixed and incubated at room temperature for 30 min. To stop the reaction, a mixture of acetonitrile:water:formic acid (94:5:1) was used to increase the volume to 1 mL, and then vortexed for 1 min, centrifuged for 15 min at 4°C, 16,000× *g*. The supernatant was sucked through a 0.22‐μm organic phase filter membrane and transferred to an HPLC (high‐performance liquid chromatography) bottle.

Quantification was carried out by Shimadzu LC‐30 AD Ultra High Performance Liquid Chromatograph (UHPLC)—Series TRIPLE QUAD 4500 Mass Spectrometer (AB Sciex, MA, USA) equipped with an electrospray ionization (ESI) source. Samples were separated in a Waters T3 column (3.0 mm × 4.6 mm; particle size 3 μm), using 0.1% formic acid, water, and acetonitrile as mobile phase. The column was thermostatically controlled at 25°C, the flow rate was set at 0.25 mL/min, and the injection volume was 5 μL. The mobile phase gradient was 95%–70%A, from 0.5 min to 2 min; 70%–95%A, from 2 min to3 min; and 95%A, from 3 min to 5 min.

Positive electrospray (ESI+) was used as the ionization source, and the method of optimization of each material parameter was carried out by capillary electrophoresis; mass spectrometer was operated in multiple reaction monitoring (MRM) mode. The monitored transitions were the following: m/z 146.0 → 118.0 for TMA–EBA; m/z 155.0 → 127.0 for d9–TMA–EBA; m/z 76.4 → 58.1 for TMAO; m/z 85.4 → 66.1 for d9–TMAO; m/z 103.9/60 for choline; m/z 162.0/102 for L‐carnitine; and m/z for 118.2/58.2 glycine betaine.

### 2.4 Metagenomic analysis of fecal samples

The total DNA of fecal samples was extracted with the FastPure Stool DNA Isolation Kit (Magnetic Bead Method), and then the extracted genomic DNA was detected by 1% agarose gel electrophoresis. DNA extract was fragmented to an average size of about 350 bp (base pairs) using Covaris M220 focused ultrasonicator for paired‐end library construction. Paired‐end library was constructed using NEXTFLEX Rapid DNA‐Seq Kit. Paired‐end sequencing was performed on Illumina NovaSeq™ X Plus at Majorbio using NovaSeq X Series 25B Reagent Kit. The originally sequenced Reads were clipped with adapters, low‐quality sequences were removed with fastp, and the Reads were aligned with the mouse genome by Burrows–Wheeler Aligner (BWA). Any Reads related to hits and their pairs were deleted. The quality‐filtered data were assembled using MEGAHIT. Contigs with a length ≥300 bp were selected as the final assembling result. A non‐redundant gene catalog was constructed using Cluster Database at High Identity with Tolerance (CD‐HIT) with 90% sequence identity and 90% coverage. Gene abundance for a certain sample was estimated by SOAPaligner with 95% identity for Short Reads Alignment. The non‐redundant gene set was compared with the non‐redundant protein (NR) and Kyoto Encyclopedia of Genes and Genomes (KEGG) databases to calculate the abundance of species and functional categories in each sample.

### 2.5 Statistical analysis

Statistical analysis was conducted on the free Majorbio Cloud platform (https://www.majorbio.com/; Shanghai Meiji Biomedical Technology Co., Ltd, Shanghai, China). The Kruskal–Wallis test followed by Tukey–Kramer post hoc test was employed to analyze differences among groups. A *p* value of less than .05 was considered statistically significant.

## 3 RESULTS AND DISCUSSION

**FIGURE 2:**
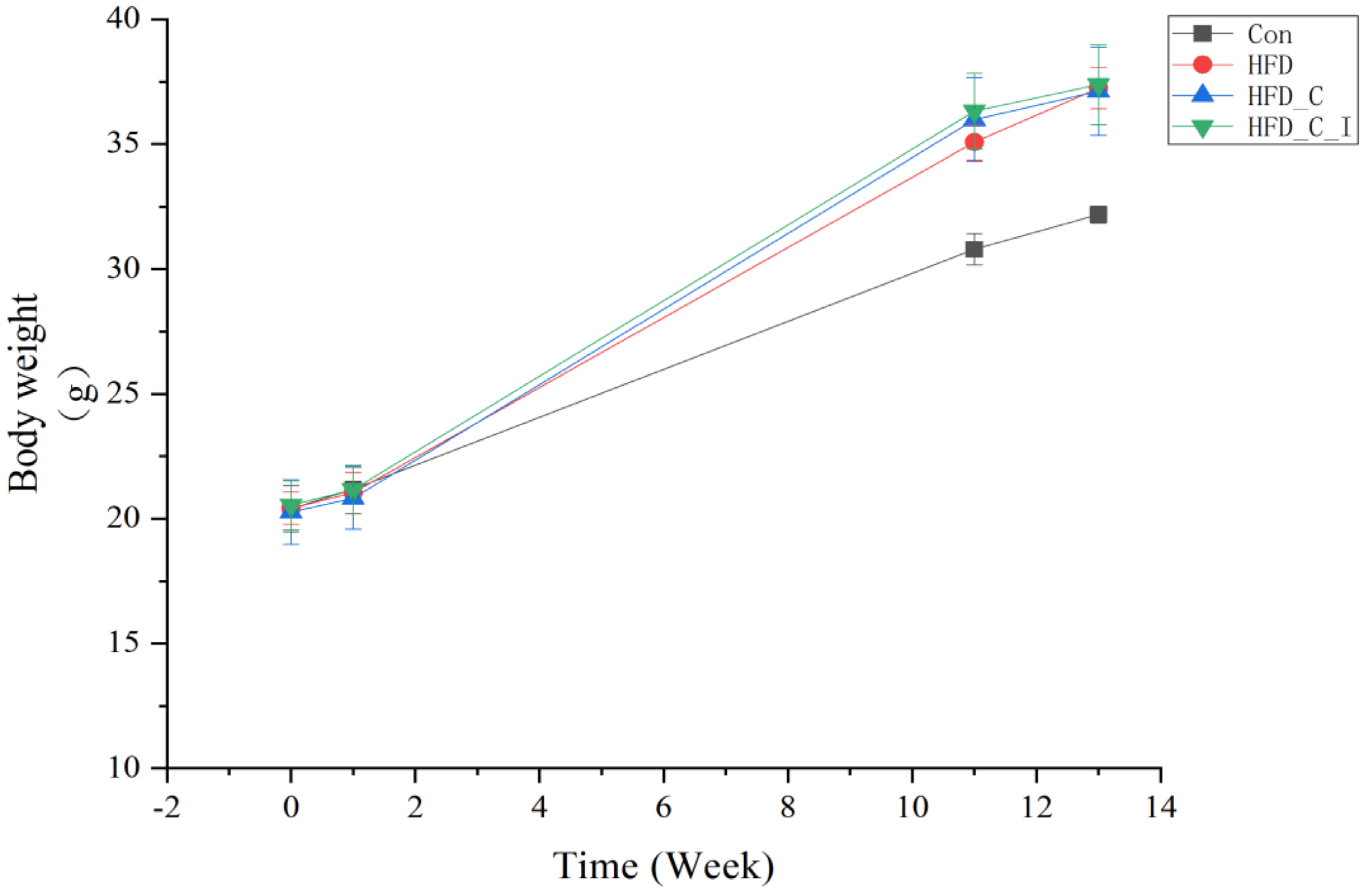
Body weight variations during dietary intervention (*n* = 5).

High‐fat diet (HFD) intervention significantly increased the body weight of mice, while the 2‐week intervention with choline and L‐carnitine or their combination with inulin had a limited effect on the body weight of high‐fat diet mice (Figure 2). The average daily water intake per mouse, as well as the intake of choline, L‐carnitine, and inulin in each group, is shown in Table S2. The results indicated that the addition of inulin increased the water intake of mice, thus slightly increasing the intake of choline and carnitine, but the differences were not statistically significant.

### 3.1 Variations of metabolites in the feces and plasma

To clarify the impact of inulin consumption on HFD‐associated elevation in cardiovascular risk, ultra performance liquid chromatography–triple quadrupole–tandem spectrometry (UPLC–QQQ–MS/MS) was used to quantify changes in TMA, TMAO, and their precursors in the feces and plasma.

**FIGURE 3:**
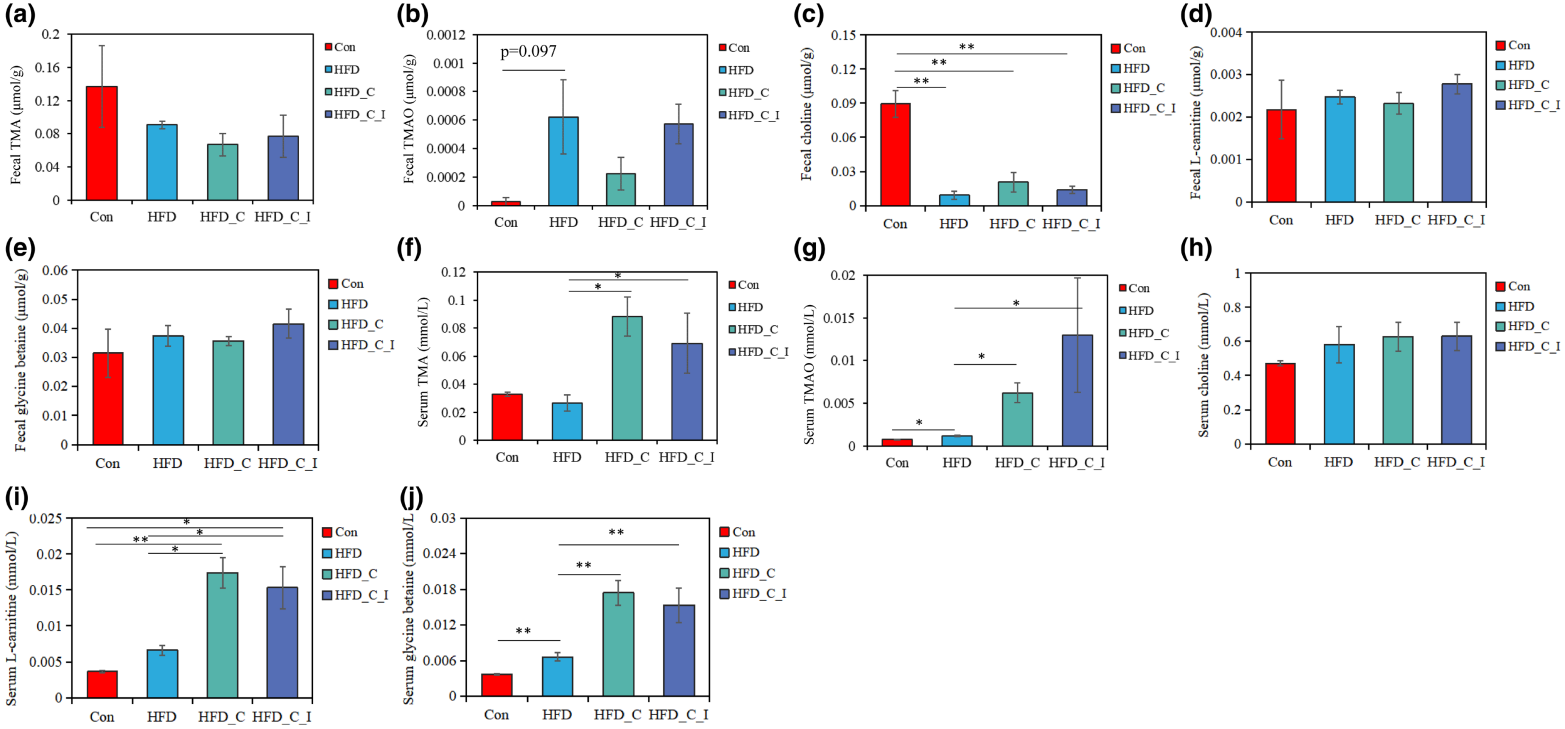
Variations of TMA, TMAO, choline, L‐carnitine, and glycine betaine in the feces (a–e) and serum (f–j). Among these compounds, the concentration of glycine betaine was presented as a relative concentration normalized to the choline signal. *.01 < *p* ≤ .05, **.001 < *p* ≤ .01. All results (*n* = 5) are reported as mean ± SEM (standard error of the mean).

As shown in Figure 3, there were no significant differences among groups in the levels of TMA, TMAO, L‐carnitine, and glycine betaine in the feces (*p* > .05). However, mice fed a high‐fat diet had significantly lower choline levels in their feces compared to the control group, while choline supplementation in the HFD_C group resulted in a slight increase in fecal choline levels, but this increase was not significant (Figure 3c). Similarly, a study by Chen et al. also found that after high‐fat diet induction, the choline content in the feces of obese rats significantly decreases (Chen et al., 2018). This suggests that HFD may promote the growth of gut bacteria that can break down choline, accelerating its degradation (Yoo et al., 2021; see Section 3.2). Additionally, phosphatidylcholine plays a crucial role in lipid transport by synthesizing very‐low‐density lipoproteins (VLDL). Therefore, under high‐fat diet conditions, the demand for choline may increase, leading to enhanced absorption (Zeisel, 2000). Furthermore, further study should focus on understanding how a high‐fat diet affects choline absorption and its metabolism.

Compared to the control group, the HFD group had significantly higher levels of TMAO and glycine betaine in the blood (Figure 3g,j). Studies by Boutagy et al. and Yoo et al. supported these findings, demonstrating that HFD treatment can increase TMAO levels in the blood (Boutagy et al., 2015; Yoo et al., 2021). However, these studies did not examine changes in TMA levels. Interestingly, a study by Lin et al. found that a high‐fat diet significantly reduced gut TMA levels (Lin et al., 2016). Similarly, in the present study, a decreased trend in fecal TMA levels was observed in the HFD group compared to the control group. A possible explanation for this may be decreased colonic barrier function, leading to enhanced transport to the liver (Jaworska et al., 2017).

In addition, the supplementation of choline and L‐carnitine significantly increased the levels of TMA and TMAO in the blood (*p* < .05). However, inulin supplementation did not have a significant effect on these metabolites, which aligns with previous reports (Baugh et al., 2018; Xiong et al., 2023). These findings suggest that inulin supplementation did not inhibit TMAO formation, even when blood TMAO levels were greatly elevated by the combined HFD and choline and L‐carnitine supplementation.

### 3.2 Impact of diet intervention on gut bacterial species linked to TMAO formation

#### 3.2.1 Gut microbiota composition

**FIGURE 4:**
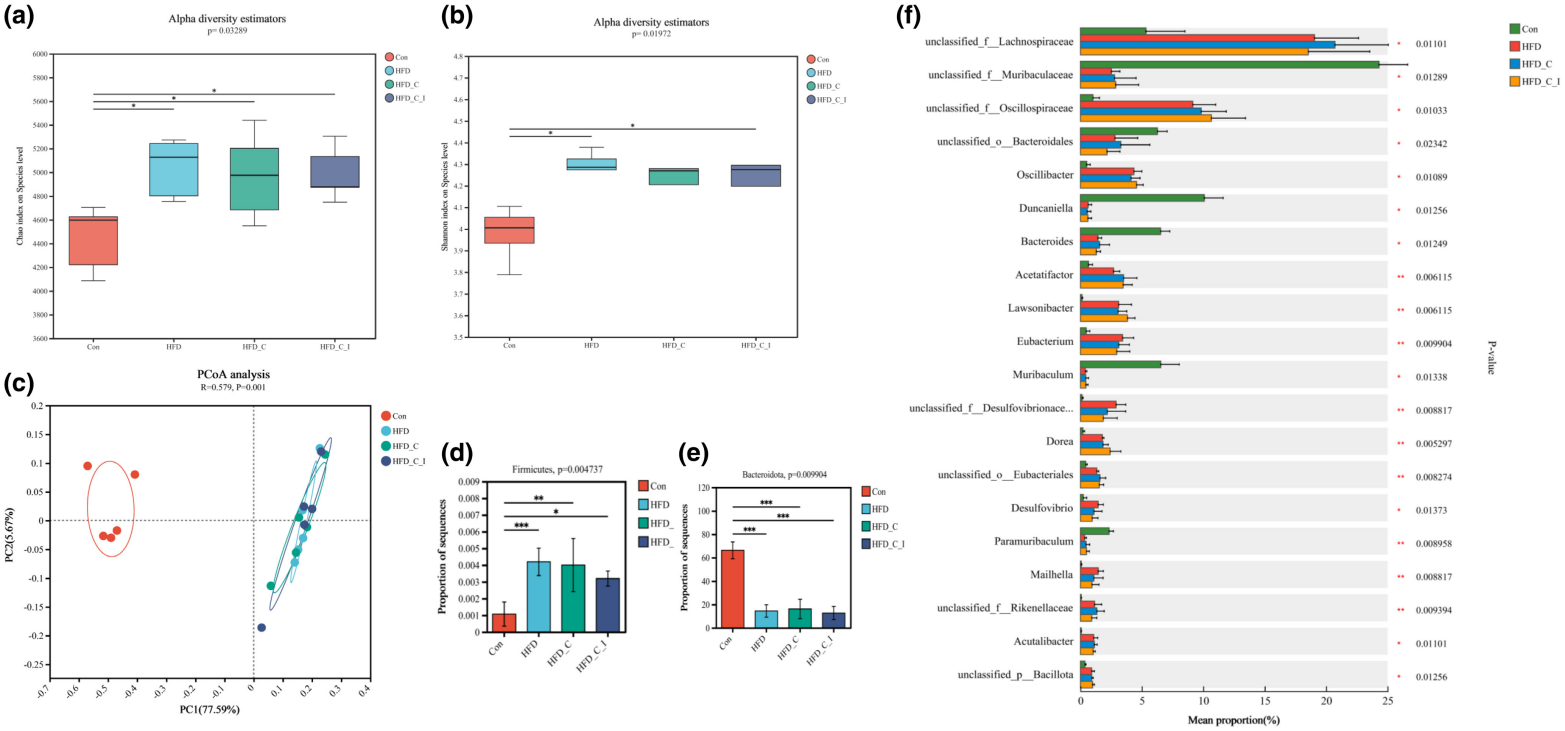
(a and b) Alpha diversity‐related indices including Chao index and Shannon index. (c) Principal coordinates analysis (PCoA) based on Bray–Curtis distance. (d and e) The relative abundance of *Firmicutes* phylum and *Bacteroidetes* phylum in difference groups. (f) Top 20 differences in bacterial genes among groups. *.01 < *p* ≤ .05, **.001 < *p* ≤ .01, ***p ≤ .001. All results (*n* = 5) are reported as mean ± SEM (standard error of the mean).

Alpha diversity reflects the richness and diversity of microbial communities within a sample, typically quantified using indices such as Chao and Shannon. As shown in Figure 4a,b, mice fed with HFD exhibited higher Chao index and Shannon index in the feces compared to mice fed with control diet, which aligns with findings from some prior studies (Wang et al., 2020; Xiao et al., 2017; Zhu et al., 2023). In contrast, Dalby et al. (2017) found that HFD treatment led to a reduction in gut α‐diversity in mice (Dalby et al., 2017). Possible reasons may be associated with variations in the choice of control diet and selection of animal strains (Wang et al., 2020). A meta‐analysis by Bisanz et al. (2019) indicated that the impact of HFD interventions on α‐diversity is not consistent, with considerable heterogeneity observed in the direction of effects. Moreover, the correlation between dietary fat intake and *α*‐diversity in gut microbiota was found to be non‐significant (Bisanz et al., 2019). However, choline and L‐carnitine or inulin supplementation did not produce significant alterations in these diversity indices. This finding is consistent with a study conducted by Xie et al., wherein they reported that choline supplementation for a duration of 14 days did not yield significant alterations in the Chao and Shannon indices (Xie et al., 2023). This lack of significant impact may be attributed to the relatively short intervention period of 2 weeks.

To evaluate the effect of diet intervention on gut microbiota composition, principal coordinates analysis (PCoA) was employed. As shown in Figure 4c, mice fed with HFD had a notable difference in microbiota composition. Compared to the changes from control diet to HFD, the supplementation of L‐carnitine and inulin showed limited effects. Many studies reported that inulin had a pronounced effect on gut microbiota composition. A possible reason may be associated with the intervention duration that is only 2 weeks. Moreover, after removal of the control group, inulin treatment also induced a significant alternation in gut microbiota composition (Figure S1).

Previous studies have indicated that HFD treatment exhibits a pronounced increase in the fecal bacterial ratio between *Firmicutes* and *Bacteroidetes* (Bisanz et al., 2019). As illustrated in Figure 4d,e, HFD treatment significantly elevated the relative abundance of *Firmicutes* while reducing that of *Bacteroidetes*. Furthermore, *Firmicutes* phylum encompasses numerous bacteria associated with TMA formation, including *Anaerococcus*, *Clostridium*, *Desulfitobacterium*, *Emergencia*, and *Streptococcus* (Jameson et al., 2018; Ramireddy et al., 2021). Therefore, an analysis of the variations in bacterial genes among these groups was undertaken. As shown in Figure 4f, significant differences were observed between the control group and the two HFD groups. Specifically, HFD treatment increased the relative abundances of unclassified *Lachnospiraceae*, unclassified *Oscillospiraceae*, *Oscillibacter*, *Acetatifactor*, *Lawsonibacter*, *Eubacterium*, unclassified *Desulfovibrionaceae*, *Dorea*, unclassified *Eubacteriales*, and *Desulfovibrio*. Conversely, it reduced the relative abundances of unclassified *Muribaculaceae*, unclassified *Bacteroidales*, *Duncaniella*, and *Muribaculum*. Some of those findings align with previous reports that HFD treatment correlates with elevated abundances of *Lachnospiraceae*, *Oscillibacter*, *Acetatifactor*, *Lawsonibacter*, *Dorea*, and *Desulfovibrionaceae* (Guo et al., 2020; Tang et al., 2024; Wang et al., 2020; Zhang, Shang, et al., 2021). Among these, *Desulfovibrio*, *Eubacterium*, and *Dorea* demonstrate the capacity to produce TMA (Borton et al., 2023; Fu et al., 2020).

#### 3.2.2 TMA formation‐associated bacterial species

**FIGURE 5:**
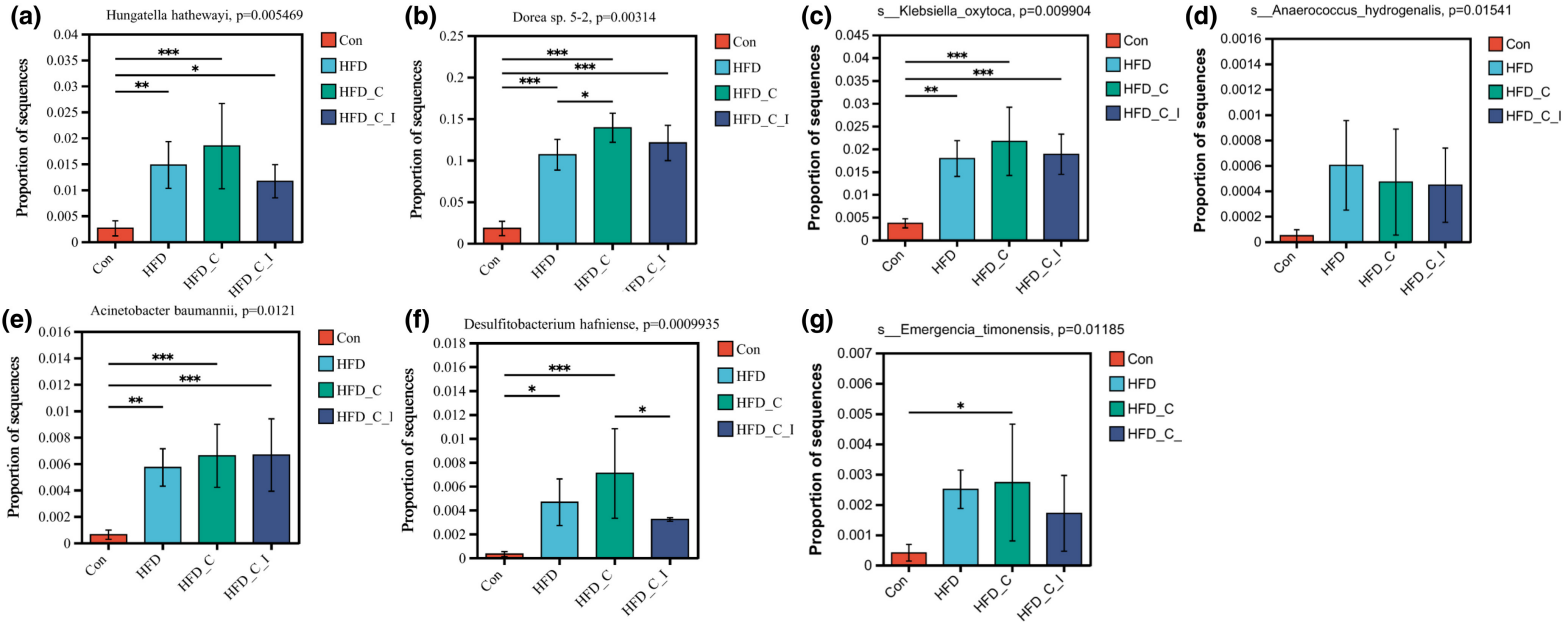
The relative abundances of bacterial species, including (a) *Hungatella hathewayi*, (b) *Dorea* sp. *5‐2*, (c) *Klebsiella oxytoca*, (d) *Anaerococcus hydrogenalis*, (e) *Acinetobacter baumannii*, (f) *Desulfitobacterium hafniense*, and (g) *Emergencia timonensis*. *.01 < *p* ≤ .05, **.001 < *p* ≤ .01, ***p ≤ .001. All results (*n* = 5) are reported as mean ± SEM (standard error of the mean).

Due to the variability in TMA production capacity among different species within the same genus, further comparisons of differences in bacterial species among groups were subsequently conducted. The differences between groups in bacterial species linked to TMA formation are shown in Figure 5.

The bacterial species *Hungatella hathewayi* (previously classified as *Clostridium hathewayi*), *Dorea* sp. *5‐2*, *Klebsiella oxytoca*, and *Anaerococcus hydrogenalis* have garnered attention for their production of choline TMA‐lyase (cutC) and its activating enzyme C/D (cutD), pivotal in the conversion of choline to TMA (Dalla Via et al., 2019; Rath et al., 2017; Romano et al., 2015). *Acinetobacter baumannii* is recognized for its significant role in the oxidation of L‐carnitine to TMA, while *Desulfitobacterium hafniense* exhibits the capacity to convert glycine betaine to TMA. Furthermore, *Emergencia timonensis* is implicated in the conversion of the conversion of γ‐butyrobetaine to TMA (Rajakovich et al., 2021). Despite the findings by Yoo et al. (2021) suggesting that HFD can influence choline conversion to TMA (Yoo et al., 2021), our discovery provides further support for a novel perspective: HFD potentially activates multiple pathways involved in TMA production, consequently elevating levels of TMA or TMAO in the bloodstream.

Supplementation of choline and L‐carnitine in the HFD group resulted in a notable increase in the prevalence of *Dorea* sp. *5‐2*, while inulin supplementation reduced the relative abundances of *D. hafniense* (Figure 5e). In addition, Figure S2 shows the top 20 bacterial species affected by inulin supplementation, but they are not directly involved in TMA formation. These findings suggest that the consumption of inulin may have only modest effects on the composition of bacterial populations involved in TMA production.

#### 3.2.3 TMA formation‐associated functional genes

**FIGURE 6:**
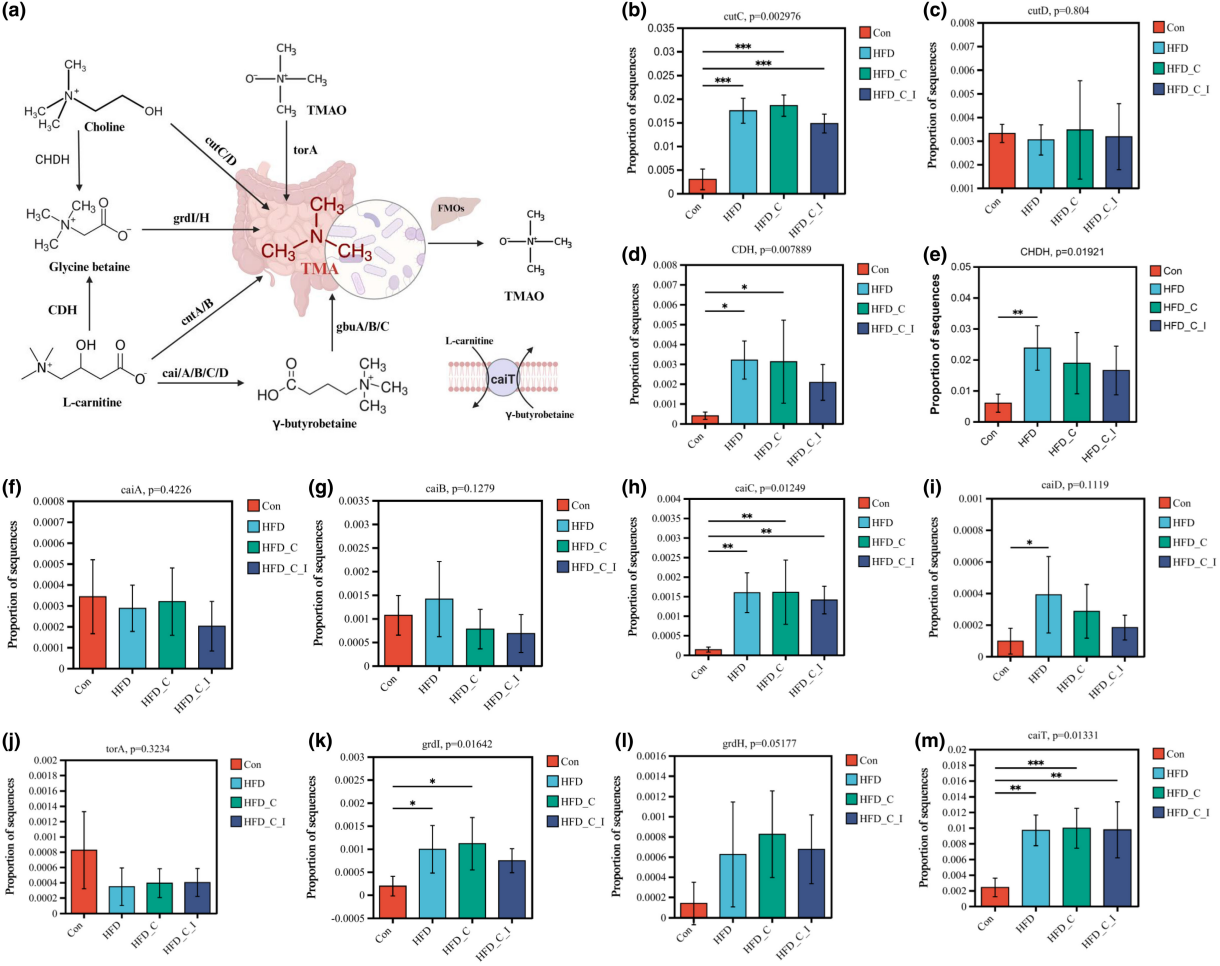
(a) The formation pathways of TMA and involved enzymes. (b–m) The relative abundances of TMA formation‐related genes, including cutC, cutD, CDH, CHDH, caiA, caiB, caiC, caiD, grdI, grdH, caiT, and torA. *.01 < *p* ≤ .05, **.001 < *p* ≤ .01, ***p ≤ .001. All results (*n* = 5) are reported as mean ± SEM (standard error of the mean).

Given the current limited identification of intestinal bacteria types involved in TMA production, it is imperative to assess the impact of dietary intervention on TMA formation by analyzing the abundance of genes associated with TMA formation. Figure 6a illustrates the pathways associated with TMA formation and highlights the corresponding enzymes involved in each pathway. First, dietary choline and L‐carnitine can be metabolized into TMA through cutC/D (Ramireddy et al., 2021) and carnitine monooxygenase (cntA/B) (Zhu et al., 2014), respectively. Alternatively, choline and L‐carnitine may undergo a conversion process, potentially involving their transformation into glycine betaine. Choline dehydrogenase (CHDH) and L‐carnitine dehydrogenase (CDH; Meadows & Wargo, 2015) are implicated in this conversion, followed by hydrolysis to TMA facilitated by betaine reductase (grdI/H; Day‐Walsh et al., 2021). Moreover, L‐carnitine can be initially metabolized into the intermediate product γ‐butyrobetaine through L‐carnitine CoA‐transferase (caiA/B/C/D; Day‐Walsh et al., 2021), subsequently converted into TMA by the γ‐butyrobetaine utilization enzymes (gbu; Dwidar et al., 2023). In addition, the L‐carnitine/γ‐butyrobetaine antiporter, caiT, also significantly contributes to L‐carnitine degradation. Interestingly, acquired through dietary intake it can undergo reduction to TMA within the colon via TMAO reductase (torA) produced by specific bacterial strains. Therefore, we examined the gene expression levels responsible for the synthesis of these enzymes in fecal samples, as depicted in Figure 6b–m.

The HFD treatment exhibited a significant effect on the expression of key functional genes implicated in metabolic pathways, including cutC, grdI, CDH, caiC, caiD, and caiT. Moreover, an upward trend in the expression levels of grdH, caiA, and caiD was discerned in fecal samples obtained from mice fed with HFD, in comparison to those on a control diet. Unfortunately, the presence of genes cntA and cntB was undetectable in fecal samples, likely attributable to the low abundance of their associated bacterial species in the feces (Rajakovich et al., 2021). Additionally, insights from a study by Koeth et al. (2014) suggest that the γ‐butyrobetaine‐associated pathway may serve as a prominent route for gut microbial metabolization of L‐carnitine in murine models (Koeth et al., 2014). However, our efforts to explore the genetic landscape were hindered by the absence of gbu gene cluster information within the current Kyoto Encyclopedia of Genes and Genomes (KEGG) database, posing a significant challenge to metagenomic analyses of these genes. Even though, the prevalence of *Emergencia timonensis*, a microbial species harboring gbu genes (Buffa et al., 2022; Rajakovich et al., 2021), exhibited an increase within the fecal bacteria of mice subjected to the HFD intervention, suggesting the HFD treatment had the potential to convert γ‐butyrobetaine to TMA. Overall, these findings have revealed that HFD can enhance the bacterial species containing genes linked to the conversion of choline→TMA, choline→glycine betaine→TMA, L‐carnitine→glycine betaine→TMA, and L‐carnitine→γ‐butyrobetaine→TMA, offering supplementary mechanistic insights into how HFD contributes to the elevation of blood TMAO levels.

Consistent with the observed impact on blood TMAO levels, as illustrated in Figure 6b–m, inulin did not significantly affect the abundance of functional genes associated with TMA formation. This further demonstrates that inulin does not exhibit an inhibitory effect on TMAO formation in mice, even with elevated blood TMAO levels caused by a high‐fat, high‐choline, and carnitine diet. This is possibly due to the limited effect of inulin supplementation on gut bacteria that contain the functional genes associated with TMA formation, as shown in Figures 5 and 6.

## 4 CONCLUSION

In summary, our findings revealed a close relationship between HFD‐elevated TMAO levels and the increased abundance of gut bacteria containing TMA formation‐associated genes. However, consistent with several previous reports, this study did not observe a significant effect of supplementation on reducing TMAO formation or the abundance of bacteria related to TMA formation. Even with a diet of HFD combined with choline and L‐carnitine, the blood TMAO levels in mice reached high levels. However, the limitations of this study include the lack of comparison between different prebiotics and the exploration of how inulin‐shaped gut bacterial species or metabolites affect TMAO formation.

## AUTHOR CONTRIBUTIONS

**Xin Wang:** Data curation (equal); investigation (equal); writing – original draft (equal). **Xiaoyi Hu:** Investigation (equal); writing – review and editing (equal). **Weiwei He:** Conceptualization (equal); supervision (equal); visualization (equal); writing – original draft (equal); writing – review and editing (equal). **Jun‐Yi Yin:** Supervision (equal); writing – review and editing (equal).

## CONFLICT OF INTEREST STATEMENT

The authors declare no conflict of interest.

## Supporting information


Data S1.

## Data Availability

The data that support the findings of this study are available from the corresponding author upon reasonable request.
