# Supplementary material for: Inulin does not affect trimethylamine N‐oxide formation in mice with a high‐fat diet combined with choline and L‐carnitine
Source: Food Sci Nutr. 2024 Sep 15;12(11):8968–77. doi: 10.1002/fsn3.4420 (PMC11606906; doi:10.1002/fsn3.4420)
Supplement: Supplementary file 1 — Data S1. [file FSN3-12-8968-s001.docx]

**Supplementary tables and figures**

**Table S1 The nutritional compositions of control diet and the high-fat diet**

| Compositions | Carbohydrate | Protein | Fat |
| --- | --- | --- | --- |
| Control diet (g/kg) | 660.00 | 208.00 | 40.00 |
| High-fat diet (g/kg) | 255.61 | 262.32 | 348.90 |

**Table S2 Daily intake of food, water, choline, L-carnitine, and inulin in the last two weeks**

| Components | Con | HFD | HFD_C | HFD_C_I |
| --- | --- | --- | --- | --- |
| Chow diet (g/day) | 3.55±0.25^a^ | 2.62±0.22^b^ | 2.12±0.26^b^ | 2.02±0.14^b^ |
| Water (g/day) | 3.64±0.28^c^ | 6.83±0.61^a^ | 4.5±0.81^b^ | 5.09±0.74^b^ |
| Choline (mg/day) | 7.11±0.50^a^ | 6.73±0.57^a^ | 51.07±9.19^b^ | 56.11±8.16^b^ |
| L-carnitine (mg/day) | 0 | 0 | 45.6±8.21^a^ | 50.9±7.4^a^ |
| Inulin (g/day) | 0 | 0 | 0 | 0.25±0.04 |

Different letters indicate a significant difference (p < 0.05) between groups


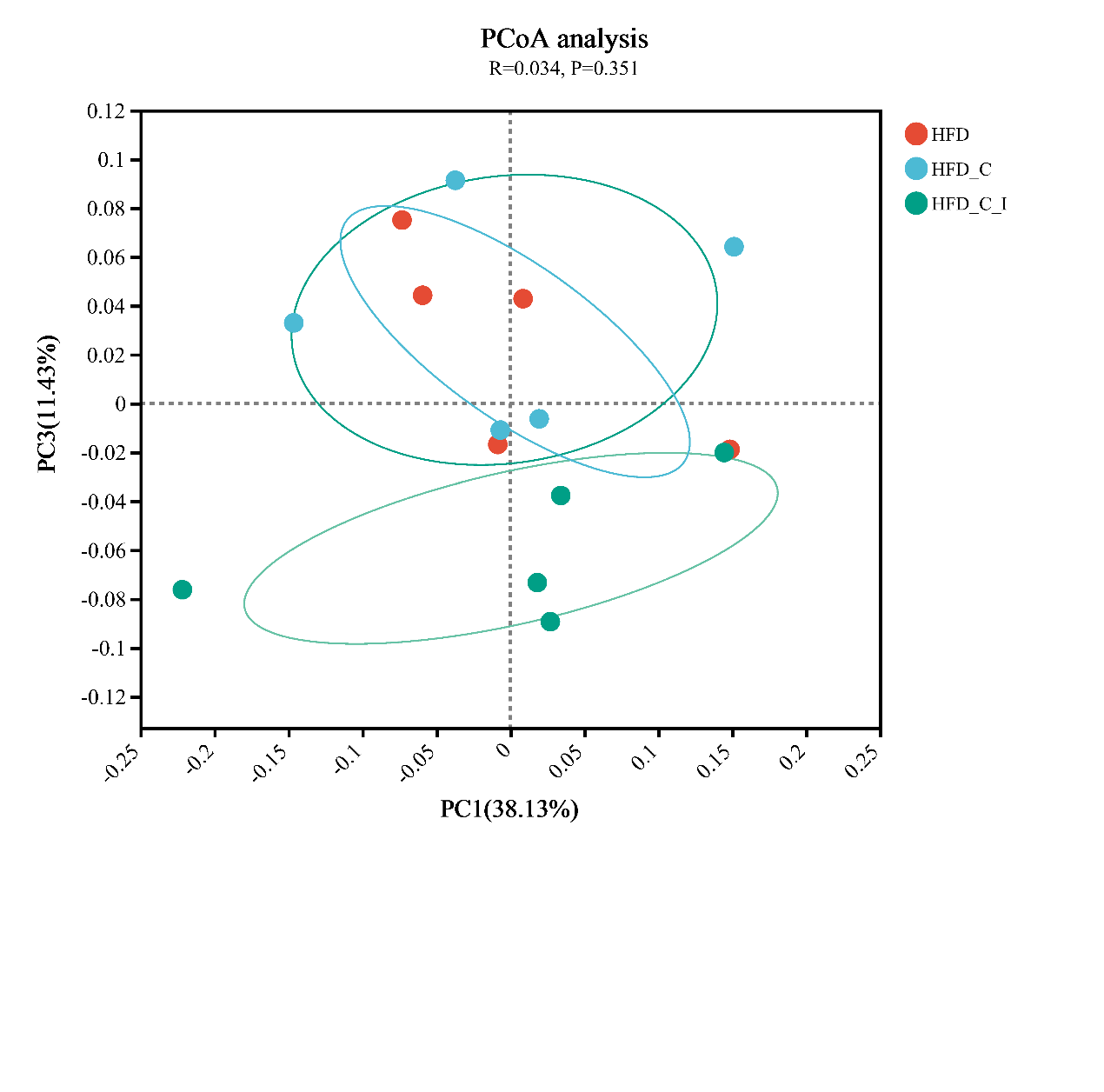


Fig. S1 PCoA analysis based on Bray-Curitis distance after removal of the control group.


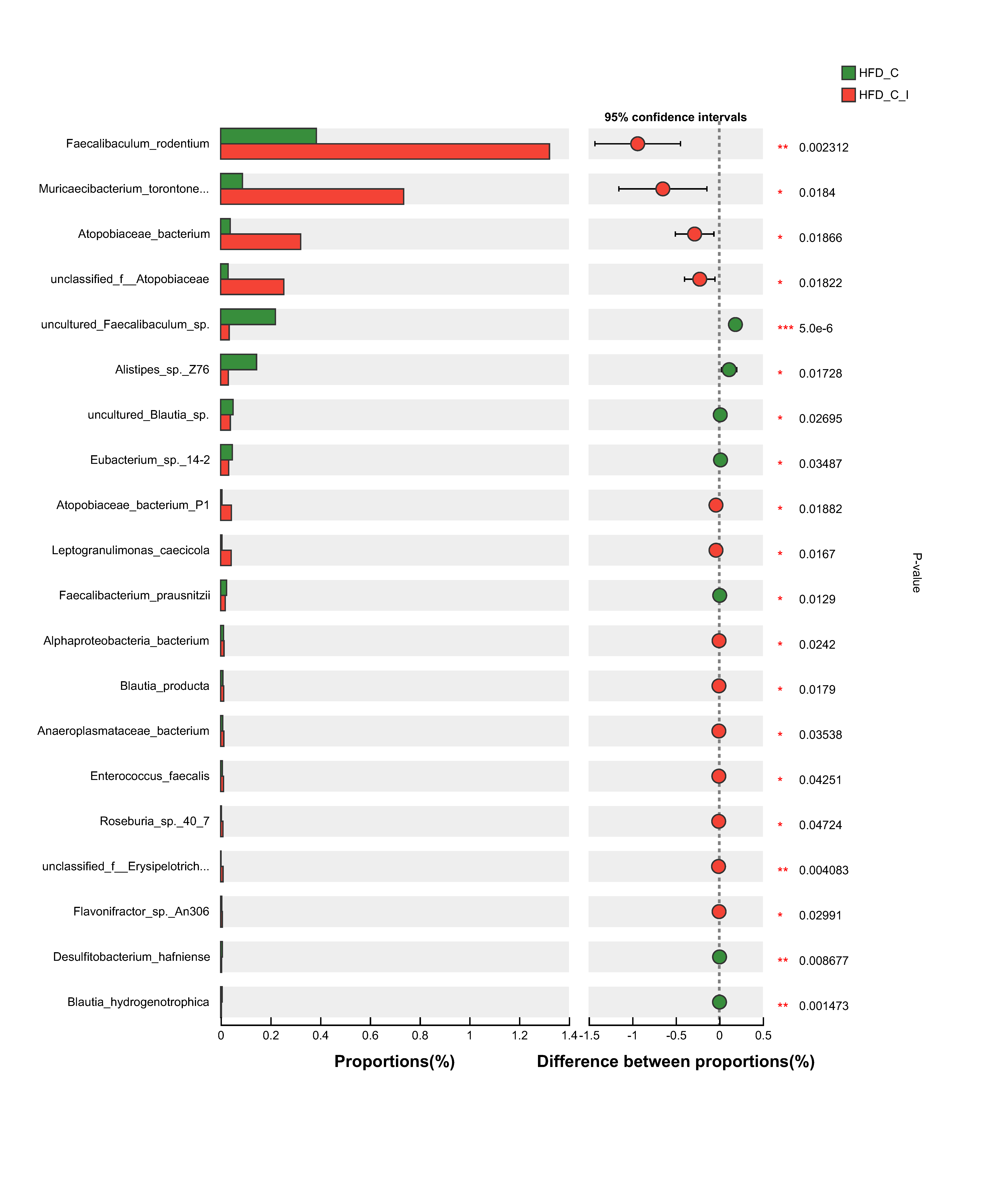


Fig. S2 Impact of inulin supplementation on the relative abundances of bacterial speceis in the feces
